# Limited enzymatic hydrolysis of green coffee protein as a technique for preparing new functional food components

**DOI:** 10.1007/s13197-022-05646-3

**Published:** 2022-12-14

**Authors:** Mostafa Ali, Harshadrai Rawel, Michael Hellwig

**Affiliations:** 1 Department of Food Technology, Faculty of Agriculture, Kafrelsheikh University, Kafrelsheikh, Egypt; 2 Institute of Nutritional Science, University of Potsdam, Arthur-Scheunert-Allee 114-116, Nuthetal, 14558 Potsdam, Germany; 3 Chair of Special Food Chemistry, Faculty of Chemistry and Food Chemistry, Technische Universität Dresden, Bergstraße 66, 01062 Dresden, Germany

**Keywords:** Enzymatic hydrolysis, Green coffee bean protein, Functional properties

## Abstract

Valorisation of side-streams in food production has become an important booster for increased sustainability in food production. The objective of this work was to study and improve the functional properties of green coffee (GC) protein. Extraction of defatted GC meal by using PVPP slightly increased protein yield and significantly decreased the amount of covalently and non-covalently bound CQA, therefore decreasing the antioxidant activity of the meal. Peptic hydrolysis at pH 1.5 led to a significantly higher degree of hydrolysis (DH) than at pH 3. Sodium dodecylsulfate polyacrylamide gel electrophoresis (SDS-PAGE) showed that the molecular weights of peptides of GC protein hydrolysates were in the range of 11–60 kDa, while peptides were in the range of 500–5000 Da using matrix-assisted laser desorption/ionization-time of flight mass spectroscopy (MALDI-TOF MS). Additionally, the enzymatic hydrolysis significantly improved the antioxidant activity of the GC protein. Finally, the results suggest that enzymatic hydrolysis with pepsin is an effective technique to provide bioactive compounds. The works presented in our manuscript may help in further exploiting the potential use of green coffee beans for food, cosmetic or pharmaceutical industry.

**Supplementary Information:**

The online version contains supplementary material available at 10.1007/s13197-022-05646-3.

## Introduction

In order to improve the sustainability of food production, it is necessary to valorise also side-streams of production earlier named “waste”. The global coffee production has reached 10 million tons with *C. arabica* (5.4 million tons per year) and *C. robusta* (4.6 million tons per year) as the most important species. Most of the coffee was produced in Brazil (2.2 million tons) followed by Vietnam (1.8 million tons) (USDA 2021). Not the whole of harvested green coffee goes into roasting and food production because of lacks in quality. Coffee oil pressed from green coffee may find expedient applications in the cosmetic and pharmaceutical industries (Moulin et al. 2022). Green coffee bean has sometimes been suggested as a source of bioactive compounds (Castro et al. 2018), but proteins, which abound in the press cake of oil production, and especially bioactive peptides have rarely been paid attention to (Ribeiro et al. 2021).

The coffee plant belongs to the family of Rubiaceae and genus of *Coffea*, with more than 100 species (Rubayiza and Meurens 2005) of which *Coffea arabica* and *Coffea robusta* are the two main species (Ramalakshmi et al. 2008). Green *C. arabica* seeds contain 8.5–12% proteins and 3.6−5.5% total phenolics (Ali et al. 2012). Green coffee (GC) bean is also a main source of chlorogenic acid (CQA) with three isomers, dicaffeoylquinic acid (diCQA) with three isomers and feruloylquinic acid (FQA) with three isomers (Ali et al. 2012). The most abundant coffee proteins are the 11S storage proteins, whose basic structure involves 3 to 6 monomers. Under reducing conditions, the disulfide bonds in 11S monomers break to release the α–acidic (~ 33 kDa) and β–basic (~ 24 kDa) subunits (Acuña et al. 1999; Ali et al. 2012).

In the literature, the extraction of GC proteins has been conducted with different methods that resulted in different colors of the extracts. Light yellow to dark green colors are produced from the interaction between CQA and amino compounds under alkaline conditions (Ali et al. 2012; Namiki et al. 2001). In addition, brown color, which is produced from enzymatic oxidation of CQA and its interaction with proteins, was also observed.

Many of animal proteins have excellent functional and organoleptic properties, but their production cost is very high compared to plant proteins. The low solubility of plant protein is the problem that makes its functionality unsatisfactory. Enzymatic hydrolysis intensively changes the functional properties of plant proteins and increases their solubility, depending on some structural changes: a decrease of molecular mass, the release of ionizable groups, and disappearance of hydrophobic regions. This may result in a higher peptide yield, better protein utilization and enhanced antioxidant activity, solubility, and digestibility of the protein (Ali 2019b; Daud et al. 2013; Kim and Yoon 2020; Mokni Ghribi et al. 2015; Yathisha et al. 2022).

Therefore, the present work aimed to optimize and apply enzymatic hydrolysis for the protein of Brazilian green coffee beans. With this approach, peptide-polyphenol mixtures were obtained, and they were chemically characterized by using reversed phase high pressure liquid chromatography (RP-HPLC) and sodium dodecylsulfate polyacrylamide gel electrophoresis (SDS-PAGE) methods in addition to matrix-assisted laser desorption/ionization-time of flight mass spectroscopy (MALDI-TOF MS). Enzymatic hydrolysis, especially at pH 1.5, significantly enhanced solubility and antioxidant activity of the extracted protein. We show that enzymatic hydrolysis with pepsin is an effective technique to provide bioactive compounds which may be exploited in food, cosmetic or pharmaceutical industry.

## Materials and methods

### Materials

Brazilian green coffee (GC) bean (*Coffea arabica)* was obtained from Green Coffee Company (Germany) and kept at − 20 °C. Pepsin (EC 3.4.21.1) and 2,2'-azino-bis(3-ethylbenzothiazoline-6-sulfonic acid (ABTS) were purchased from Sigma Aldrich, Germany. Bovine serum albumin (BSA), polyvinylpolypyrrolidone (PVPP) and trolox (TE, 6-hydroxy-2,5,7,8-tetra-methylchroman-2-carboxylic acid) were bought from Serva (Heidelberg, Germany), and DHAP (2,5-dihydroxy actetophenone) was obtained from Bruker Daltonik GmbH, Germany.

### Methods

The experimental scheme of the present work is shown in the supporting information (Figure S1).

### Sample preparation

The frozen GC beans were milled using Retsch Ultra Centrifugal Mill ZM 200 (Haan, Germany). The milling was done at 15,000 rpm speed, and afterwards, the GC powder was defatted with *n*-hexane (1:10 w/v, flour/solvent) at room temperature for 6 h three times. After air drying overnight at room temperature, the powder was kept at − 20 °C until analysis.

### Extraction of GC bean protein

GC bean protein was extracted by 0.04% ascorbic acid solution with and without PVPP as reported by Ali et al. (2012). Forty g of GC meal and 0 and 10 g of PVPP were dispersed in 400 mL of ascorbic acid solution. After that, the combinations were stirred at room temperature for 3 h and centrifuged at 4000 × g for 30 min at room temperature. The supernatants were then dialyzed (dialysis tube, 6–8 kDa) against distilled water for 24 h at 4 °C and the retentates were freeze dried.

### Determination of protein content of extracts

Protein content was measured by Bradford (1976) method using BSA as a standard.

### Covalently and noncovalently bound chlorogenic acid (CQA)

The amount of CQA covalently and non-covalently bound to the proteins and hydrolysates was measured according to the method described in Ali (2019a). Four mg of sample was dispersed in 1 mL of 8 M urea solution, and after that the protein was precipitated by addition of 20% trichloroacetic acid (50:50). Then, the precipitate was dissolved again in 1 mL of 8 M urea. Shimadzu Reversed-phase high-performance liquid chromatography (RP-HPLC, Kyoto, Japan) with a Perfectsil column (C8 300 ODS, 150 × 4.6 mm, 5 μm) at 37 °C was used to evaluate the content of covalently and non-covalently bound CQA. 0.1% Trifluoroacetic acid (v/v) and acetonitrile were (A) and (B) eluents, respectively with the gradient: B (10−18%), 22 min; B (18−80%), 8 min; B (80%), 3 min; B (80−10%), 2 min; and B (10%), 7 min. The injection volume was 50 μL and the total run time was 42 min. Calibration for 5-CQA was performed at 325 nm.

### Determination of the antioxidant capacity of proteins and hydrolysates using trolox equivalent antioxidative capacity (TEAC) assay

The antioxidant capacity was determined as mentioned in Ali et al. (2013). The extracted protein and hydrolysates were prepared in 5 mM PBS buffer pH 7.2–7.4 and mixed with ABTS^+^ solution and stored for 6 min at room temperature. Afterward, the absorbance was recorded at 730 nm using a spectrophotometer (Pharmacia Biotech, England). The data are calculated as nmol TE/mg protein.

The next analyses of the protein extract were carried out on the sample extracted with ascorbic acid with PVPP. The choice of this sample was due to the low CQA content that usually affects protein extraction and changes the protein color depending on the interaction between the protein and CQA during the extraction.

### Enzymatic hydrolysis of green coffee bean protein

The hydrolysis process was done according to Ali (2019b). The protein was extracted as described above, using ascorbic acid and PVPP, then the protein content was measured, and pH was modified using 2 M HCl to 1.5 and 3.0 and the temperature was set at 34 °C. The ratio of pepsin enzyme to protein was 1:100 (w enzyme/w protein). Samples were taken after 0 and 10 min, 1, and 3 h and the pH adjusted to 7.6–8.0 to deactivate pepsin. After that, the solutions were freeze-dried and stored at − 20 °C until analysis.

### Determination of degree of hydrolysis (DH)

The protein content was measured according to Bradford (1976) and the DH of protein was estimated as the percentage of the protein content after hydrolysis to the total protein content before hydrolysis.

### Determination of solubility of the hydrolysates

As described previously (Ali 2019b), 1 mg of extracted protein and hydrolysates was dissolved in 1 ml of distilled water with pH 7. The obtained suspension was mixed and exposed to ultrasound for 5 min, then centrifuged at 9250 × g for 10 min at 4 °C and the protein content of supernatants before and after centrifugation was tested according to Bradford (1976).

### Determination of free amino groups content of hydrolysates by trinitrobenzenesulfonic acid (TNBS) method

The free amino groups content of GC hydrolysates was evaluated using the method of Ali et al. (2013). Isoleucine in the range from 20 to 100 nM was used to prepare the calibration curve.

### Determination of protein content of hydrolysates using RP-HPLC

Reversed Phase–High Performance Liquid Chromatography was used to study the protein content. Four mg of GC protein and hydrolysates were well mixed with 1 mL of PBS buffer, pH 7.2, then analysed by HPLC as written in details in Ali et al. (2018).

### Estimation of the molecular weight (MW) of hydrolysates using SDS-PAGE

Sodium Dodecyl Sulfate—Polyacrylamide Gel Electrophoresis (SDS-PAGE) protocol as outlined by Ali et al. (2013) was used to estimate the MW of hydrolysates. Sigma Marker in the molecular range of 14–96 kDa was employed as a marker and the Quantity One® 1-D analysis Software (Bio-Rad Laboratories, Italy) was employed to determine the MW and intensity of protein bands.

### Estimation of the molecular weight (MW) of hydrolysates using MALDI-TOF MS

Matrix Assisted Laser Desorption Ionization—Time of Flight Mass Spectrometry (MALDI- TOF MS) technique based on Ali et al. (2013) was used to estimate the MW of peptides in the hydrolysates by dissolving 1 mg of GC protein and hydrolysates in 1 mL of 0.1% trifluoroacetic acid/acetonitrile (50%, v/v). DHAP matrix was used to cover the samples and measured using MALDI–TOF MS (Bruker Daltonik GmbH, Germany) apparatus, and evaluated with the Bruker Daltonics Flex Analysis software.

### Statistical analysis

The results were statistically examined by SPSS program version 18. Data of *P* ≤ 0.05 were considered statistically significant (Kumar et al. 2016).

## Results and discussion

### Characterization of green coffee (GC) meal bioactivities after extraction using ascorbic acid and polyvinylpolypyrrolidone (PVPP)

Chlorogenic acid (CQA) can interact covalently and non-covalently with GC protein during extraction and produces colored products (Ali et al. 2012). The digestibility of these products is low, moreover, the consumers do not accept them. Polyvinylpolypyrrolidone (PVPP) is a water-insoluble polymer that adsorbs polyphenols via hydrogen bonding and other weak forces. It is thought that they interact with polyphenols via H bonds between their CO–N linkages and phenol groups (Laborde et al. 2006). Further PVPP has been used to remove polyphenols from natural products such as the extract of black tea (Ranatunge et al. 2017). The effect of ascorbic acid is its reducing power, and it was applied in this study in order to reduce intermittently formed chlorogenic acid quinones that could react at nucleophilic sites on proteins. Therefore, in this study, the GC protein was extracted using 0.04% of ascorbic acid with and without PVPP to test the effect of both substances on the protein content, covalently and non-covalently bound CQA content and antioxidant capacity and the obtained results are presented in Table 1.

**Table 1:** Characterization of GC meal bioactivities extracted using ascorbic acid and PVPP

Properties	Extraction materials	
	Ascorbic acid with PVPP	Ascorbic acid without PVPP
SDS-PAGE for proteins under reducing conditions		
Intensity of bands (AU)		
Band 1: 32 kDa	2163.44 ^b^ ± 17.06	2574.38 ^a^ ± 61.08
Band 2: 22 kDa	2152.72 ^b^ ± 11.45	2577.28 ^a^ ± 23.03
Protein content (g/100 g DW)	2.33 ^a^ ± 0.35	2.08 ^a^ ± 0.17
Free CQA (mg/100 g DW)	367.70 ^b^ ± 79.16	675.44 ^a^ ± 86.18
Covalently bound CQA (mg/100 g DW)	24.59 ^b^ ± 2.77	61.95 ^a^ ± 10.62
Antioxidant capacity (mM TE/100 g DW)	13.77 ^b^ ± 0.45	17.86 ^a^ ± 0.42

Values followed by the same small alphabetic letter a, b, c and d in a row are not significantly different at *P* ≤ 0.05

The results indicated that the use of ascorbic acid with PVPP enhanced the protein quality compared to extraction without PVPP. Using PVPP seems to be important to bind CQA and prevent its reaction with protein during the extraction (Laborde et al. 2006; Ranatunge et al. 2017). Moreover, ascorbic acid helps avoiding oxidation of CQA to quinones that can interact with amino and thiol groups of proteins and produce CQA-protein conjugates, which principally affect the color and quality of protein. The results of SDS-PAGE show two main bands, α (acidic) and β (basic), with molecular weights (MW) ~ 32 and 22 kDa, respectively. There were observable differences in the intensity of the bands, where the intensity of the α band decreased from 2574.38 to 2163.44 while the intensity of the β-band decreased from 2577.28 to 2152.72 when PVPP was used, respectively (Table 1). This result may be attributed to the possibility of PVPP to decrease the interaction between coffee protein and CQA during the extraction. Moreover, PVPP improved protein extraction where the amount of extracted protein was increased from 2.08 to 2.33 g/100 g DW when PVPP was used. However, this increase was not significant. Concerning the content of covalent and non-covalent CQA, results reveal that the powder extracted using ascorbic acid in the absence of PVPP had about twice the amount of bound and unbound CQA compared to that PVPP had been used for (Table 1).

This finding is very interesting in the field of protein extraction from beans that have high phenolic content such as coffee beans and sunflower seeds. These data are in accordance with Bridi et al.(2014) who found that no polyphenols were found in tea extracts after PVPP treatment. Therefore, PVPP can be used with ascorbic acid to decrease the amount of phenolic compounds bound to protein during the extraction and produces a protein with light color and higher solubility, which can be used to supply cereal foods or a great protein source to produce weaning foods. Besides, it could help the developing countries to reduce the protein malnutrition problem. The antioxidant capacity of GC meals was 13.77 and 17.86 mM trolox equivalents (TE)/100 g DW for samples extracted with ascorbic acid with and without PVPP, respectively (Table 1). The high antioxidant capacity of meal extracted in the absence of PVPP can be explained by the high free and bound CQA content compared to that extracted in the presence of PVPP, also for compounds produced during processing.

### Characterization of GC protein hydrolysates

#### Degree of hydrolysis (DH)

**Fig. 1:**
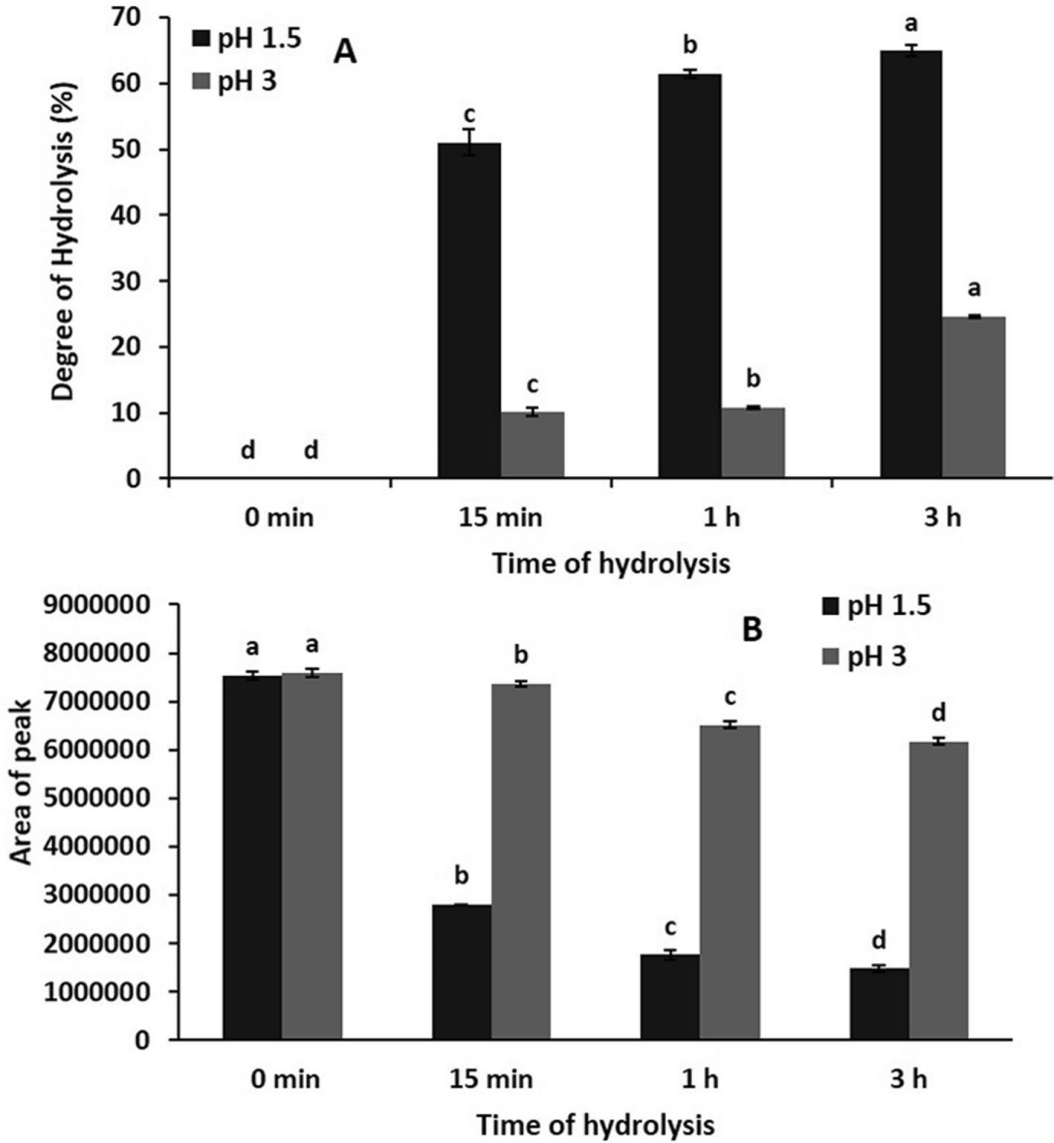
Influence of hydrolysis time and different pH on the degree of hydrolysis of GC bean protein (**A**) and the protein content using RP-HPLC (**B**). Values followed by the same small alphabetic letter a, b, c and d in a row are not significantly different at *P* ≤ 0.05

After enzymatic hydrolysis of protein, it is necessary to calculate the DH. The effect of pH 1.5 and 3 and the hydrolysis periods 0, 15 min, 1 and 3 h, on the DH were examined and the obtained findings are shown in Fig. 1A. The findings revealed that with increasing hydrolysis period, the DH of protein at both pH was significantly increased. As displayed also in Fig. 1A, the DH at pH 1.5 was significantly higher than that at pH 3, where the percentage of DH increased from 0 at 0 min to 65% after 3 h hydrolysis at pH 1.5, while at pH 3 the percentage increased to only 25% when hydrolysis was run from 0 to 3 h. The high DH at pH 1.5 compared to pH 3 may be related to the optimal conditions of the enzyme. The same observation was detected during the hydrolysis of different proteins such as soya, rice bran, faba bean and fish using different enzymes, papain, alcalase, enzyme cocktail, and pepsin (Ahmadifard et al. 2016; Ali 2019b; Chabanon et al. 2007; Daud et al. 2013; Dryaková et al. 2010; Karamac and Rybarczyk 2008; Wisuthiphaet et al. 2015; Yathisha et al. 2022). At the end, it could be summarized that the degree of protein hydrolysis is associated with the conditions of hydrolysis, pH and time.

#### The area under curve using the RP-HPLC method

The change in native GC protein content, after enzymatic hydrolysis was evaluated by RP-HPLC using the change in the area under the curve at 280 nm and the results are outlined in Fig. 1B. The results indicated that the protein content was significantly decreased with increasing incubation period. Incubation at pH 1.5 showed the highest reduction in the area under curve, where the value was reduced by 80.5% after 3 h. By contrast, it was reduced by 18.6% after hydrolysis for 3 h at pH 3. These results are in agreement with Ali (2019b) who found that the content of faba protein, calculated as area under curve, was decreased when the protein had been treated with pepsin at pH 1.5 and 3 for different periods.

### Characterization of the molecular weight of the hydrolysates

#### Estimation of the molecular weight of the hydrolysates using Sodium dodecylsulfate polyacrylamide gel electrophoresis (SDS-PAGE)

**Fig. 2:**
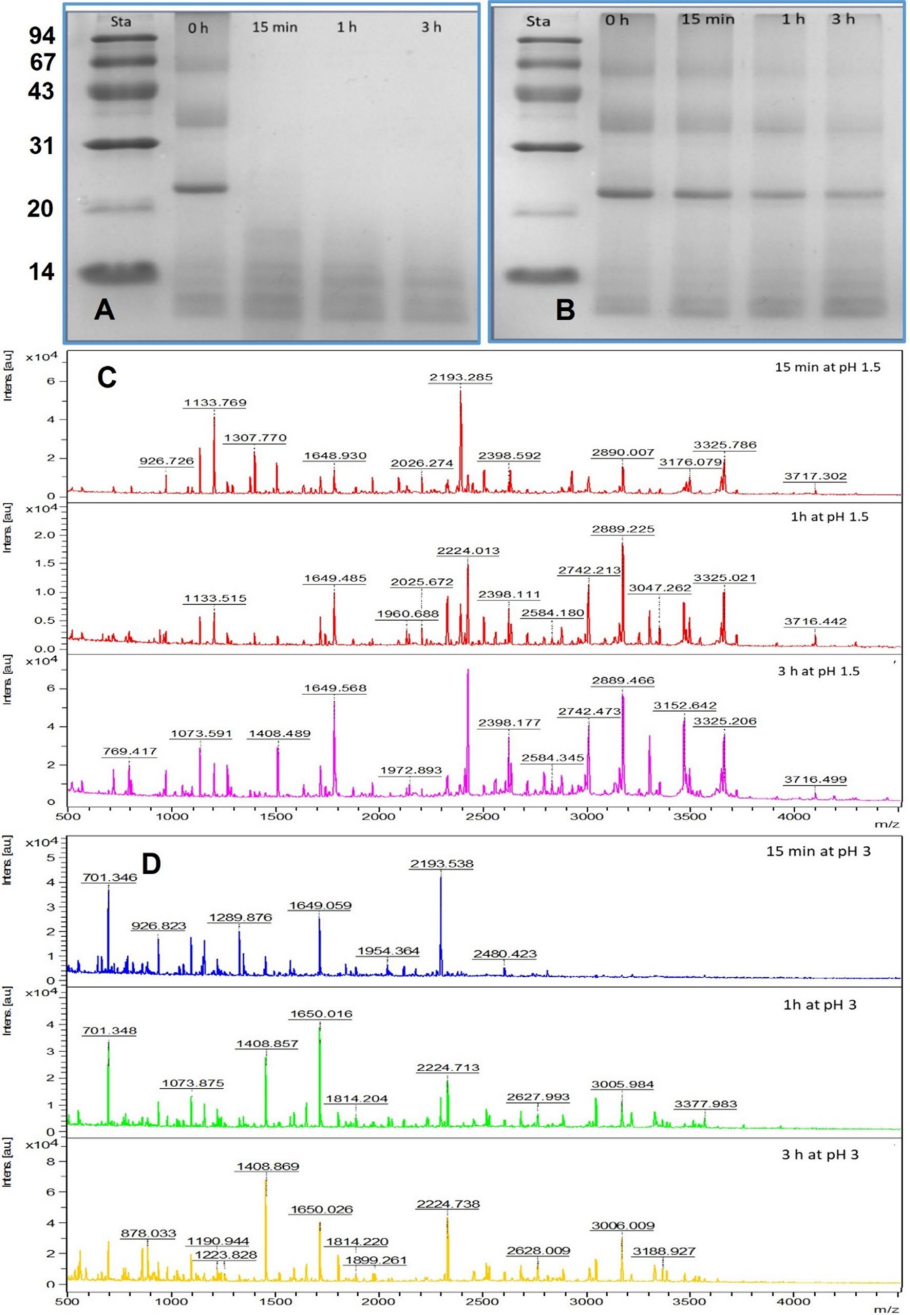
SDS-PAGE profiles of GC protein after hydrolysis at pH 1.5 (**A**) and pH 3 (**B**) and molecular weights (Da) of the hydrolysates using MALDI-TOF MS after hydrolysis at pH 1.5 (**C**) and pH 3 (**D**) for different times. Sta, molecular weights of marker. Line 1 in each Figure displays the MW of the standards, lines 2, 3, 4, and 5 show the hydrolysates found after hydrolysis at pH 1.5 and 3 for 0, 15 min, 1 and 3 h

The MW of an 11S coffee storage protein is 300–400 kDa and after treatment with non-reducing materials such as SDS or urea can be separated to produce six subunit pairs with MW of 55–60 kDa, each pair being linked by a disulfide bond. The breaking of these disulfide bonds under reducing conditions gives two subunits (α-acidic and β-basic) with MW ~ 33 and 24 kDa, respectively (Ali et al. 2012; Campos et al. 2022; Fukshima 2001). Polyacrylamide gel electrophoresis under denaturing conditions was performed to study the effect of the pepsin enzyme treatment, at two different pH (1.5 and 3) and for 0, 15 min, 1, and 3 h, on GC protein breakdown. The findings are presented in Fig. 2A and B and Table 2. SDS-PAGE separated hydrolysed proteins into subunits and polypeptides.

**Table 2:** Molecular weights (kDa) and intensity of bands of GC protein after hydrolysis at pH 1.5 and 3 for different times

Hydrolysis time	Lane	MW (kDa)	pH 1.5		pH 3	
			Peak Int	Relative Qty	Peak Int	Relative Qty
0 min	2	58–60	1401.56	11.81	1447.25	12.77
		35–36	1509.94	12.82	1558.31	14.39
		23–24	2118.81	51.80	2308.75	57.63
		12–13	1345.56	11.52	1648.56	15.20
		11–12	1400.00	12.06	N.D	N.D
15 min	3	58–60	1184.88	8.78	N.D	N.D
		35–36	1233.88	8.94	N.D	N.D
		23–24	1816.13	4.44	N.D	N.D
		17–18	N.D	N.D	1205.06	14.90
		14–15	1113.81	8.33	1543.56	19.11
		13–14	1208.06	9.05	1660.38	21.15
		12–13	1391.75	10.41	1791.00	22.94
		11–12	1450.63	10.05	1737.75	21.89
1 h	4	58–60	937.81	29.93	N.D	N.D
		35–36	1173.19	29.78	N.D	N.D
		23–24	1509.19	19.73	N.D	N.D
		14–15	1200.56	4.49	1370.25	21.93
		13–14	1326.50	5.01	1602.75	26.31
		12–13	1531.31	5.78	1627.81	26.77
		11–12	1498.44	5.28	1613.94	25.00
3 h	5	58–60	777.19	5.26	N.D	N.D
		35–36	955.88	6.42	N.D	N.D
		23–24	1328.56	21.51	N.D	N.D
		14–15	1189.56	7.92	1229.06	21.18
		13–14	1413.94	9.66	1447.56	25.51
		12–13	1638.81	29.30	1501.56	26.52
		11–12	1641.94	19.94	1539.19	26.79

N.D means the band was not detected

According to Rawel et al. (2005) and Ali (2012), the GC protein was separated using SDS-PAGE into two bands with MW ~ 22 and 32 kDa. The SDS-PAGE profile of GC protein (Figs. 2A and B, line 2) showed 5 bands with MW ranged from 11 to 59.5 kDa. Results given in Fig. 2A and shown in Table 2 reveal that the intensity of bands formed at pH 1.5 gradually reduced and some bands vanished. Besides, other new bands, identified as peptides, appeared compared to pH 3 which exhibited also a reduction in the bands intensity, but only two bands disappeared (12 and 11 kDa) (Fig. 2B and Table 2). GC protein displayed many protein bands not fully digested by pepsin at pH 3 compared to pH 1.5. The bands corresponding to non-hydrolyzed protein in GC continued until 3 h of incubation at pH 3 while at pH 1.5, they only stayed until 15 min.

#### Estimation of the molecular weight of hydrolysates using matrix-assisted laser desorption/ionization-time of flight mass spectroscopy (MALDI-TOF MS)

The GC protein hydrolysates formed after enzymatic hydrolysis at pH 1.5 and 3 were estimated using MALDI-TOF MS to find the MW of peptides (Fig. 2C and D), which represents an excellent method reflecting the hydrolysis of proteins. Moreover, this technique was also used to identify small peptides which cannot be analyzed with SDS-PAGE. The obtained results showed the MW of peptides between 500 and 5000 Da (Fig. 2C and D). GC protein hydrolyzed at pH 1.5 and 3 for 15 min showed many peptides with high intensity which decreased and some of them vanished with increasing hydrolysis time. In general, the findings in the Figures show that GC protein was hydrolyzed at pH 1.5 better than at pH 3, because at pH 1.5 the number of peptides produced was higher, as well as the intensity of peaks. In contrast, GC protein incubated with pepsin at pH 3 did not hydrolyze well even after 3 h. These results are in accordance with results of Ali (2019b) who reported that the faba bean protein hydrolyzed with pepsin at pH 1.5 was more affected than at pH 3. Finally, these results support the DH, SDS-PAGE, and RP-HPLC results.

### The covalently and non-covalently bound CQA content

**Fig. 3:**
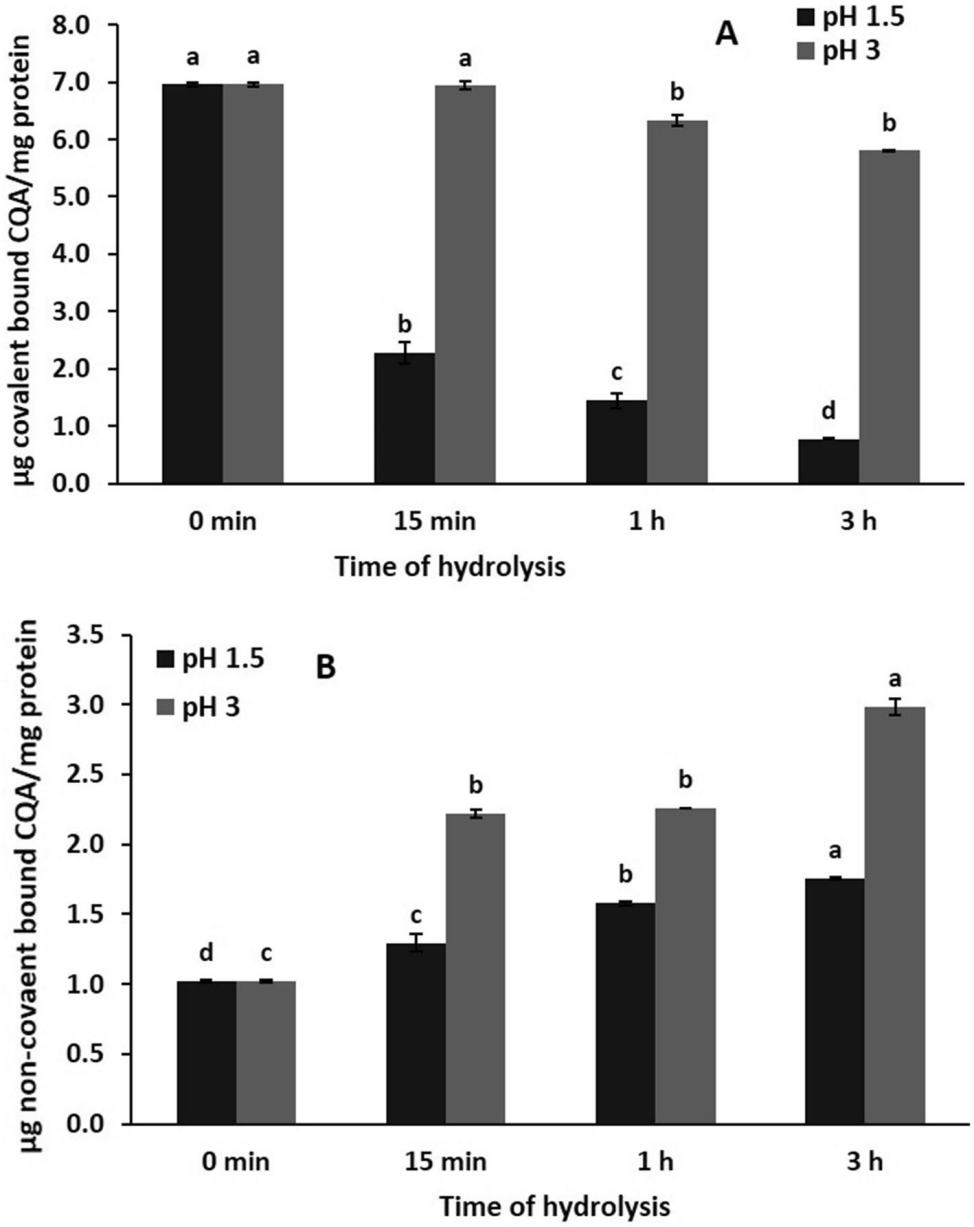
Covalently (**A**) and non-covalently (**B**) bound CQA content of GC protein hydrolysates after hydrolysis at pH 1.5 and pH 3 for different times

The content of CQA of GC protein is one of the main components during extraction. CQA can interact covalently and non-covalently with coffee protein during extraction and produces colored products (Ali et al. 2012; Rawel et al. 2005). The non-covalently bound CQA can be released by enzymatic hydrolysis. Therefore, the free and bound CQA contents of the hydrolysates were measured, and the data are presented in Fig. 3A and B. The results show that the content of bound CQA gradually decreased at two pHs while the content of free CQA (non-covalent) was gradually increased with an increasing induction period. These results can be explained by the action of proteolytic enzymes that leads to the release of non-covalently bound CQA (e.g., bound by hydrophobic interactions) from protein. This was already shown for trypsin in a previous work (Ali et al. 2013). From the results in the two figures, it can also be noticed that the decrease and increase in free and bound CQA were significant at *P* ≤ 0.05. In addition, the hydrolysis at pH 1.5 showed a significantly higher effect compared to hydrolysis at pH 3. The high effect for pH 1.5 may be related to the optimal pH for pepsin.

### The functional properties of GC protein hydrolysates

#### The content of free amino groups (FAG)

**Fig. 4:**
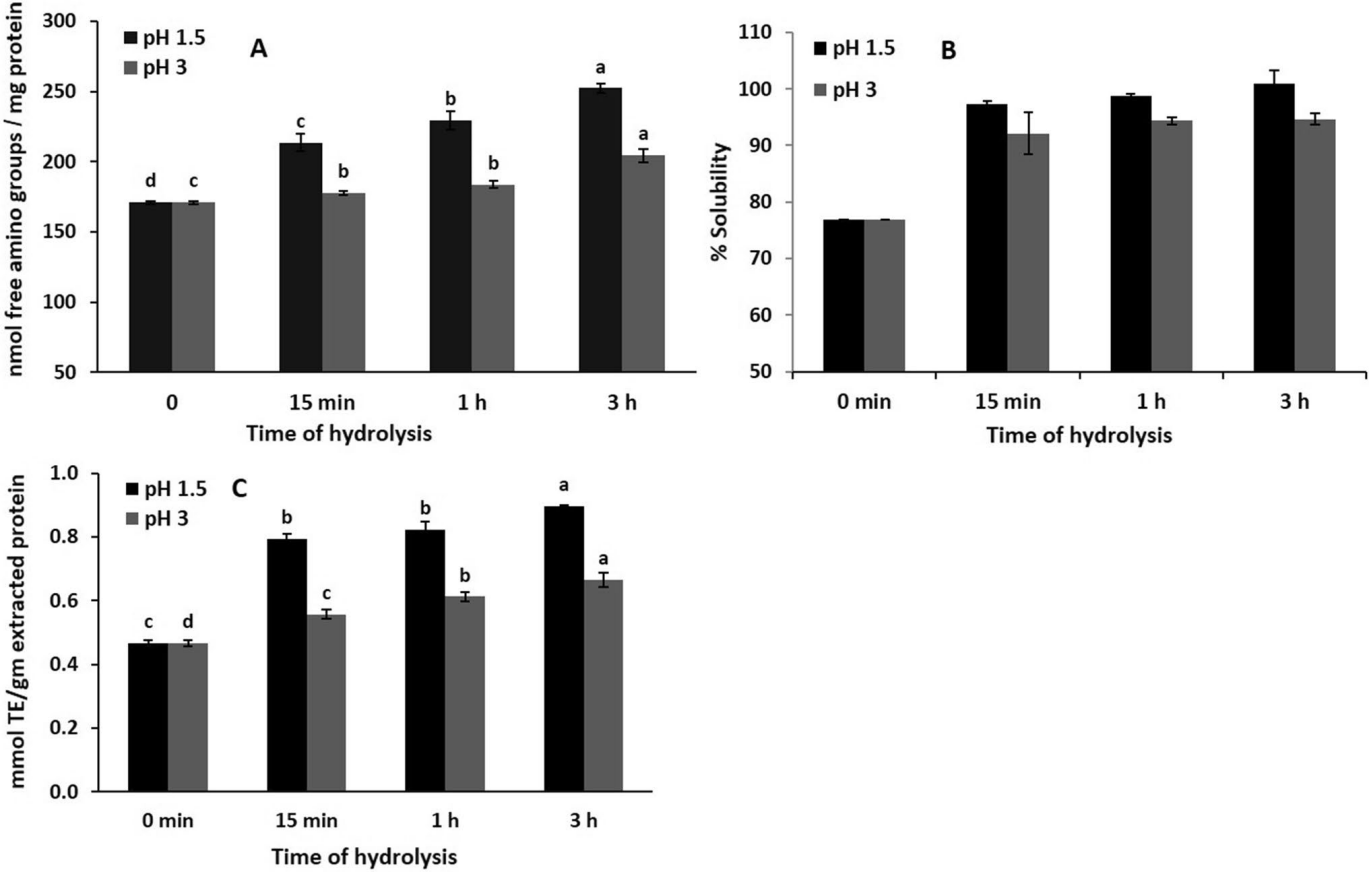
Influence of hydrolysis time and different pH on free amino group content, protein solubility, and the antioxidant activity of GC protein hydrolysates

Figure 4A shows the influence of enzymatic hydrolysis of GC protein, at different pH and incubation periods, on the FAG content. The results show that the FAG content was increased at pH 1.5 and 3 with increasing hydrolysis period. As shown in Fig. 4A, the content of FAG was 213.60 nmol/mg protein after 15 min of incubation at pH 1.5 and significantly increased with increasing incubation time to reach 252.40 nmol/mg protein after 3 h, whereas at pH 3 it was 177.60 nmol/mg protein after 15 min of incubation and gradually increased to 204.40 nmol/mg protein after 3 h. The data confirm that the enzymatic hydrolysis of GC protein shows a positive correlation between the content of FAG and both pH and the hydrolysis period.

#### Solubility of GC hydrolysates

The solubility of protein is considered one of the most important functional properties and is very essential for food applications. The solubility of hydrolysates, produced at different pH and times, compared to protein at zero time was measured and the results are presented in Fig. 4B. The solubility of hydrolysates at two pHs was significantly higher than that of the protein at zero time, especially at pH 1.5, where the value was 77% for undigested protein and between 92 and 100% for hydrolysates. Moreover, results also showed that the solubility of hydrolysates at pH 1.5 was significantly higher than at pH 3. These results can be explained by the action of proteolytic enzymes that break down peptide bonds, releasing peptides and amino acids that have improved solubility compared to whole proteins. The solubility of chickpea protein was increased after treatment with alcalase (Mokni Ghribi et al. 2015).

#### Antioxidant capacity of hydrolysates by trolox equivalent antioxidative capacity (TEAC) assay

The antioxidant capacities of GC protein hydrolysates were compared using the TEAC assay (Fig. 4C). The antioxidant capacity changed over time and hydrolysates formed at pH 1.5 showed a significantly higher antioxidant capacity than those formed at pH 3. The antioxidant capacity was higher at 15 min and then significantly increased with incubation time. The value of antioxidant capacity was 0.466 mmol TE/g protein at zero time hydrolysis, after that the values increased for all hydrolysates formed, from 0.793 to 0.896 and from 0.557 to 0.664 mmol TE/g protein after hydrolysis from 15 min to 3 h at pH 1.5 and 3, respectively. As stated by Graszkiewicz (2007) and Lin et al. (2011), lysozyme and whole egg white proteins hydrolyzed by trypsin and alcalase exhibited strong antioxidant activity. Also, incubation of whey, soya, dairy protein concentrates, skim milk proteins, fish meat of red Tilapia, and faba bean protein with different enzymes improved the antioxidant capacity (Ali 2019b; Conway et al. 2012; Daud et al. 2013; Dryaková et al. 2010; Yang et al. 2011).

The hydrolysis of protein produces high content of free amino groups. These amino acids should probably exhibit good antioxidant capacity in hydrolysates (Dryakova´ et al. 2010; Xiong 2011). The data in this work show that enzymatic hydrolysis significantly enhances the antioxidant capacity of GC protein. The antioxidant activity of proteins could be explained by their sulfur, aromatic and basic amino acids which can donate H to free radicals (Je et al. 2005; Rajapakse et al. 2005).

## Conclusion

The valorisation of side-streams of food production is a quite important step for the improvement of the sustainability of food production. This study gives helpful information for using enzymatic hydrolysis processes to improve the functional properties of the protein extracted from green coffee beans. It was found that the functional properties of GC protein were improved using the enzymatic hydrolysis process. GC protein could be hydrolyzed by pepsin at pH 1.5 and 3 and the degree of hydrolysis at pH 1.5 was higher than that at pH 3. Peptic hydrolysis can result in a higher amount of antioxidant peptides after only 15 min incubation. Finally, we can conclude that the enzymatic hydrolysis technique of GC protein enhanced the solubility and antioxidant capacity of protein; thus it could be exploited as novel component for food or ingredients used in the cosmetic and pharmaceutical industries to enhance its functional properties and nutritional and technological value. Finally, test model systems closer to real food processing conditions would be helpful to evaluate whether plant protein hydrolysates could be a viable alternative for other functional protein sources.

## Supplementary Information

Below is the link to the electronic supplementary material.

Supplementary file1 (DOCX 91 KB)

## Data Availability

The datasets used during the current study are available from the corresponding author on reasonable request.

## Abbreviations

BSA: Bovine serum albumin
CQA: Caffeoylquinic acid (chlorogenic acid)
DH: Degree of hydrolysis
DHAP: 2,5-Dihydroxyacetophenone
DW: Dry weight
FAG: Free amino groups
FQA: Feruloylquinic acid
GC: Green coffee
MW: Molecular weight
PBS: Phosphate-buffered saline
PVPP: Polyvinyl polypyrrolidone
TE: Trolox equivalent
TEAC: Trolox equivalent antioxidative capacity
TNBS: Trinitrobenzenesulfonic acid
TOF: Time of flight
USDA: United States department of agriculture
