# Supplementary material for: Limited enzymatic hydrolysis of green coffee protein as a technique for preparing new functional food components
Source: J Food Sci Technol. 2022 Dec 14;60(2):609–20. doi: 10.1007/s13197-022-05646-3 (PMC9873858; doi:10.1007/s13197-022-05646-3)
Supplement: Supplementary file 1 — Supplementary file1 (DOCX 91 KB) [file 13197_2022_5646_MOESM1_ESM.docx]

**Limited enzymatic hydrolysis of green coffee protein as a technique for preparing new functional food components**

**Supporting information**


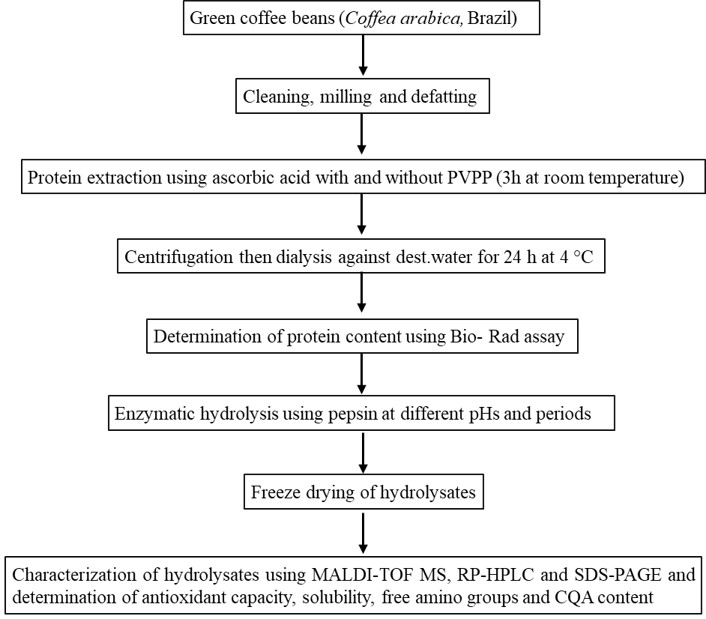


**Figure S1: Experimental scheme showing the main phases of the research.**
